# Multitask Learning for Mental Health: Depression, Anxiety, Stress (DAS) Using Wearables

**DOI:** 10.3390/diagnostics14050501

**Published:** 2024-02-26

**Authors:** Berrenur Saylam, Özlem Durmaz İncel

**Affiliations:** Computer Engineering Department, Boğaziçi University, 34342 İstanbul, Türkiye

**Keywords:** deep learning, LSTM, regression, ensemble learning, random forest, XGBoost, wearable devices, multitask learning, mental health, digital health, pervasive health, digital biomarker

## Abstract

This study investigates the prediction of mental well-being factors—depression, stress, and anxiety—using the NetHealth dataset from college students. The research addresses four key questions, exploring the impact of digital biomarkers on these factors, their alignment with conventional psychology literature, the time-based performance of applied methods, and potential enhancements through multitask learning. The findings reveal modality rankings aligned with psychology literature, validated against paper-based studies. Improved predictions are noted with temporal considerations, and further enhanced by multitasking. Mental health multitask prediction results show aligned baseline and multitask performances, with notable enhancements using temporal aspects, particularly with the random forest (RF) classifier. Multitask learning improves outcomes for depression and stress but not anxiety using RF and XGBoost.

## 1. Introduction

Smartwatches and fitness trackers are examples of wearable devices that have become popular with their ability to continuously monitor various physiological signals and record real-time temporal trends. The abundance of temporal data has intriguing prospects for tailored, context-aware applications, providing many insights into human activities, health monitoring, and behavior analysis.

One particular application area focuses on monitoring and predicting individuals’ mental health, encompassing their emotional and psychological states. Mental health is crucial in determining an individual’s sense of contentment, happiness, and overall fulfillment in life.

Mental health is a multifaceted aspect of human well-being, encompassing dimensions such as depression, anxiety, and stress. The World Health Organization (WHO) indicated that, in 2019, around 301 million people suffered from anxiety, and 280 million people lived with depression https://www.who.int/observatories/global-observatory-on-health-research-and-development/analyses-and-syntheses/mental-health/global-strategic-direction (accessed on 17 January 2024). These two factors are known to be related to mental disorders and may also be referred to as mental health conditions https://www.who.int/news-room/fact-sheets/detail/mental-disorders (accessed on 17 January 2024). Stress is another factor affecting mental and physiological problems https://www.apa.org/topics/stress/health (accessed on 17 January 2024).

To measure these three important factors for mental health, psychologists developed assessment techniques based on questionnaires. One of the most well-known scales was developed in 1995 by Lovibond and Lovibond, and it is called the Depression, Anxiety, and Stress Scale (DASS) [1]. The main contribution of this new scale is to be a combined version of separate questionnaires for each factor. Traditional approaches to mental health assessment often rely on subjective self-reports and clinical interviews, which may not capture the full spectrum of these conditions. The accurate assessment and differentiation of these constructs have been long-standing challenges in the field. In recent years, there has been a growing interest in leveraging wearable devices to monitor and predict mental health, responding to the increasing awareness of the importance of mental well-being in modern society [2].

The sensors integrated into wearable devices provide non-invasive means of collecting continuous data on individuals’ physiological and behavioral patterns. This invaluable resource enables us to explore the intricate connections between mental health and wearable-derived digital biomarkers. The most adapted devices are smartphones and smartwatches. These devices have various sensors, such as accelerometer, heart rate, electro-dermal activity (EDA), etc. Their computational capacity enables them to collect continuous data and even run machine learning models on them to extract meaningful, high-level, human-interpretable information.

Traditional machine learning algorithms, such as logistic regression, decision trees, and ensemble methods, have been extensively used to classify well-being and mental health using data collected from wearables and questionnaires [3,4]. However, many studies often overlook the temporal aspect of the data, not considering how past data impacts future mental health levels.

Integrating time-based prediction techniques with wearable devices provides a unique opportunity to capture and analyze temporal patterns, facilitating early detection and timely interventions to improve mental well-being. Deep learning architectures, including recurrent neural networks (RNNs), particularly long short-term memory (LSTMs), have emerged as powerful tools for addressing time-based prediction problems.

In this paper, we utilize the NetHealth dataset [5] and specifically focus on the prediction of students’ mental health, collected from undergraduate students at Notre Dame University spanning from Fall 2015 to Spring 2019, constituting eight waves corresponding to each semester. The dataset encompassed data from approximately 700 students during the 2015–2017 period and 300 students during the 2017–2019 period, reflecting variations in participation rates over time. This rich dataset comprised information from various modalities, including social networks, physical activity, sleep data collected from Fitbit wearable devices, and ground truth data sourced from questionnaires.

When there is a set of related but not identical tasks ${\left\{T_{i}\right\}}_{i=1}^{m}$ for *i* ranging from 1 to *m*, multitask learning endeavors to enhance the learning of a model for task $T_{i}$ by harnessing the knowledge embedded in the other tasks, either individually or as a subset of the entire set [6]. In this paper, we delve into the domain of multitask learning (MTL). This powerful machine learning paradigm enables the simultaneous modeling of multiple mental health dimensions, including depression, anxiety, and stress, in our context. By jointly training models for these tasks, we aim to enhance predictive accuracy and achieve a more comprehensive understanding of mental well-being. In our study, where different tasks in multitask supervised learning share the training data, MTL can be viewed as a generalization of multi-output regression.

Our investigation encompasses a wide spectrum of predictive techniques. We delve into deep learning methodology, specifically LSTM networks, to capture temporal dependencies in mental health data. Additionally, we incorporate conventional prediction algorithms such as random forest (RF) and XGBoost to provide a holistic view of mental health assessment. In addition, we consider their performances in the time-based (lagged) version of data. By leveraging wearable technology, MTL, and advanced predictive techniques, we aim to enhance our understanding of the complex interplay between depression, anxiety, and stress.

Our study seeks to address the following research questions:

- RQ1: What are the underlying biomarkers of mental well-being, considering factors such as stress, anxiety, and depression?
- RQ2: Are these biomarkers in line with conventional psychological studies?
- RQ3: Do time-based versions of conventional algorithms lead to improved prediction performance in the context of mental health?
- RQ4: Do further methodologies such as MTL on top of time-based models improve the final performance?

This paper is organized as follows: Section 2 provides insights into the motivation of the DASS and sheds light on the existing literature related to mental health monitoring with wearables and, most importantly, what has been done in the literature about mental health via multitask learning and considering the time aspect. In Section 3, we delve into the details of the motivation of methodology and details of the methods, such as the dataset, the missing data handling process, and the preprocessing steps undertaken before the analysis, along with the details regarding the algorithms. Section 4 presents the results of our analyses, including related biomarkers to the target variables and a wide range of performance of applied algorithms. In Section 5, we present the model performance results based on various types, from conventional to time-based and multitask. Finally, we conclude the paper with Section 6, offering interpretations, discussions, comparisons to related studies, and insights into the future of mental health prediction.

## 2. Background and Related Work

### 2.1. Motivation for Depression, Anxiety, Stress (DAS)

Lovibond and Lovibond [1], aimed to create an instrument proficient in measuring the symptomatology associated with depression and anxiety while concurrently distinguishing between these constructs. In the course of developing this instrument, they incorporated clinical and diagnostic symptoms pertaining to depression and anxiety. Symptoms shared between these disorders, such as alterations in appetite, were deliberately excluded from consideration.

Nonetheless, during the initial validation stages, factorial analyses yielded an unexpected third factor, namely “stress”. This factor, as described by the authors [1], encompasses symptoms associated with difficulty in relaxing, nervous tension, irritability, and agitation. Consequently, the first iteration of the instrument, known as the DASS-42 (a longer version consisting of 42 items), was introduced. This instrument has since become one of the most widely utilized tools worldwide for assessing affective symptoms. It comprises three distinct scales:

Depression Scale: This scale measures feelings of hopelessness, low self-esteem, and diminished positive affect.

Anxiety Scale: It evaluates autonomic arousal, musculoskeletal symptoms, situational anxiety, and the subjective experience of anxious arousal.

Stress Scale: This scale gauges tension, agitation, and negative affect.

Responses to each item in the instrument pertain to experiences within the preceding week and are categorized into four Likert responses: 0, indicating “Nothing”, to 3, signifying “Most of the time”. Subsequently, a shorter version of the DASS, now recognized as the DASS-21 [1] (Depression, Anxiety, and Stress Scale-21 items), was developed. This concise iteration includes seven selected items from each of the three scales.

### 2.2. Mental Health

The pervasive impact of mental illness on emotions, reasoning, and social interactions underscores the urgent need for early detection to enable effective prevention and intervention. Recognizing the potential of machine learning as a tool in predicting mental health, researchers employ this technology to extract valuable insights and develop intelligent systems to enhance overall well-being [3,4].

In the landscape of mental health research, the diagnosis of mental health problems necessitates a diverse repertoire of techniques, ranging from traditional interviews and questionnaires to the sophisticated monitoring of physiological signals. Common mental disorders, including schizophrenia, depression, bipolar disorder, anxiety disorders, and post-traumatic stress disorder reveal themselves through distinct characteristics and symptoms that intricately impact cognition, emotion, and behavior. This multifaceted nature demands advanced methodologies for analysis, where data mining emerges as a pivotal player in knowledge discovery within databases. In conjunction, machine learning, harnessing the power of artificial intelligence to glean insights from experiential data, becomes indispensable. Commonly employed machine learning approaches encompass supervised and unsupervised learning, providing researchers with tools to delve into complex patterns and relationships within mental health datasets [3,7].

The gaps in the literature that have been found draw attention to issues including small sample sizes, poor validation, little deep learning research, and a lack of real-world testing. Future research directions that have been suggested call for more in-depth investigation of deep learning, the collection of high-quality data, the creation of precise prediction tools, the explanation of models, the use of transfer learning, and adaptable algorithms [7].

### 2.3. Time-Based and Multitask Learning for Mental Health

In [8], researchers delved into the temporal dimension of health monitoring, specifically exploring the utilization of heart rate variability (HRV) data from wearable devices for predicting mental and general health outcomes. The research focuses on the feasibility of forecasting complex health measures, including stress, anxiety, depression, and overall well-being, based on HRV data collected over short durations. Employing advanced LSTM networks, a sub-type of deep recurrent neural networks designed for sequence analysis, the study achieves notable classification accuracies of up to 85%. The emphasis on utilizing short-duration data streams, ranging from two to five minutes, underscores the potential for real-time and efficient health predictions. This research highlights the significance of temporal dimensions in health assessment by demonstrating the capacity to derive relevant health predictions from brief periods. This maximizes the usefulness of wearable devices for ongoing health monitoring.

In another study [9], researchers introduced a machine learning model utilizing electronic health records (EHRs) to predict mental health crises continuously over 28 days. The model, developed to address the impracticality of manual review in busy clinical settings, achieves a notable area under the receiver operating characteristic curve of 0.797. A subsequent 6-month study in clinical practice demonstrates the algorithm’s value, proving effective in managing caseloads and mitigating crisis risks in 64% of cases. The model’s performance is evaluated across disorders, age groups, and data availability, revealing consistent effectiveness and highlighting key predictive features such as historical symptom severity, hospital interactions, and patient characteristics.

The study [10] explores the prediction of well-being factors, including stress, anxiety, positive affect, and negative affect, using data collected from office workers through wearable devices. The research compares the performance of machine learning algorithms, such as RF, XGBoost, and LSTM, in conventional and time-based versions. The findings highlight the significance of time-based predictions, with LSTM showing the best performance. The study identifies digital biomarkers, including personality factors, sleep-related parameters, and activity levels, as influential in predicting well-being. The results suggest practical applications in mental health monitoring, early interventions, and workplace well-being programs.

From the point of view of MTL studies, in [11], researchers propose a deep learning model for predicting human moods based on calendar events, aiming to address challenges such as skewed data and overfitting. The model incorporates an event feature extractor using LSTM and introduces MTL to enhance the prediction accuracy. The research focuses on the impact of events on mood, particularly calendar events, as they reflect user-specific situations. The proposed model outperforms existing machine learning models, with MTL helping prevent overfitting, especially on days with significant mood fluctuations. The evaluation demonstrates improvements in undertraining and prediction accuracy, highlighting the model’s effectiveness in capturing variations in mood due to different events. Limitations include the standardized participant profile, further validation with diverse populations, and improved data quality control methodologies.

In [12], the researcher introduces a MTL framework comparing three formulations: MTL deep neural networks, multitask multi-kernel learning, and a hierarchical Bayesian model for predicting mood, stress, and health outcomes. The primary focus is on personalization, allowing individuals to possess tailored models while still leveraging data from the general population. MTL significantly enhances the mood prediction performance compared to traditional machine learning models. The accuracy of MTL models, personalized for individual differences, ranges from 78% to 82%, an improvement over the 60–66% accuracy of one-size-fits-all models. The personalized MTL models outperform both single-task learning (STL) and MTL models that multitask over related outcomes (mood, stress, health).

In addition to the provided studies, there is also a focus on the application of new machine learning applications. For instance, in [13], the authors proposed an attention-based LSTM system to improve mobile emotion recognition. The system integrates behavioral and physiological signals from smartphones and wristbands, achieving an 89.2% accuracy in binary positive and negative emotion classification. The system, which uses 45 participants’ behavioral and physiological responses, is adaptable to different data signals and can handle scenarios with varying signal availability. The proposed attention mechanism enhances its ability to focus on relevant information, and its technical robustness is attributed to the combination of diverse signals and rigorous evaluation methodology.

Furthermore, survey questions have been utilized in the literature to improve model performances. For instance, in [14], the researchers used the RF algorithm and ecological momentary assessment (EMA) to predict depressive symptoms in emerging adults. The random forest algorithm achieves superior predictive accuracy, utilizing 13 out of 36 variables. Age, worry-related emotions, and a specific social exchange emotion regulation strategy are crucial predictors of severe depressive symptoms. The study uses 33 participants who engage in EMA through a mobile app, providing data on affect, ER strategies, and sociodemographic variables. The RF algorithm outperforms other models, emphasizing the importance of 13 variables in predicting depressive symptoms. The combination of EMA and ML contributes to early identification and prevention strategies.

This study focuses on the temporal aspects of integrating MTL techniques in student well-being prediction. Leveraging methods such as RF, XGBoost, and LSTM, we delve into the temporal patterns and dependencies present in the data to predict various well-being indicators, such as depression, anxiety, and stress, based on historical data. Our research simultaneously considers the interplay of these factors within a time-based and multitask framework, introducing lagged versions of both baseline and MTL algorithms that account for diverse time windows, showcasing their potential to capture temporal trends. This study extends the application of MTL to the NetHealth dataset, addressing a gap in current research. We aim to contribute to the literature by addressing research questions stated in Section 1.

## 3. Methodology

This section provides a detailed overview of our methodology, driven by the motivation to apply time-based and multitask models. We provide dataset details and highlight used modalities with target variables to provide baseline information about the aim of this study. We explain the imputation methodology since we include more than one semester’s data by imputing the missing values in the other semester’s data. We also explain data preprocessing steps before the model training and provide details about the used models.

### 3.1. Motivation for Methodologies

#### 3.1.1. Time-Based Modeling

Time-based modeling in mental health utilizes temporal data and techniques for profound insights, prediction, and management of conditions over time [15]. It is particularly valuable in the context of longitudinal studies, where data are collected over extended periods, enabling the exploration of how mental health issues evolve over time and the identification of influential factors. Time-series analysis, focusing on fluctuations in symptoms, mood, and behaviors, aids in pattern recognition and identifying triggers. Predictive models leverage historical data to forecast future mental health outcomes, potentially paving the way for proactive interventions. These models also facilitate the determination of optimal intervention timings, a crucial aspect of effective mental healthcare. Wearable technology, capable of monitoring physiological and behavioral markers, can be integrated into these models for real-time monitoring and feedback [16].

#### 3.1.2. Multitask Learning

Multitask learning (MTL) aims to train models for multiple similar tasks simultaneously [17]. Generally, it has been used to reduce overfit and improve model accuracy [18]. In the context of mental health, instead of training separate models for each mental health aspect (depression, anxiety, stress), one can use MTL to build a single model that predicts all three simultaneously. This approach recognizes that different aspects of mental health, such as depression, anxiety, and stress, are interconnected and can benefit from joint modeling, considering their interdependencies. Additionally, it can help identify shared risk factors or protective factors across different mental health conditions, contributing to a deeper understanding of the complexities of mental health. MTL can potentially enhance mental health assessment, intervention, and support by leveraging the synergy between multiple mental health-related tasks and domains.

### 3.2. Dataset

In this study, we utilize the NetHealth dataset http://sites.nd.edu/nethealth/ (accessed on 27 January 2024) [5], which was gathered from undergraduate students at Notre Dame (ND) University between the Fall of 2015 and the Spring of 2019. This dataset covers eight distinct periods (waves), each corresponding to a semester. Initially, it included data from approximately 700 students from 2015 to 2017, but this number dropped to 300 during the 2017–2019 period due to decreased participation.

**Figure 1:**
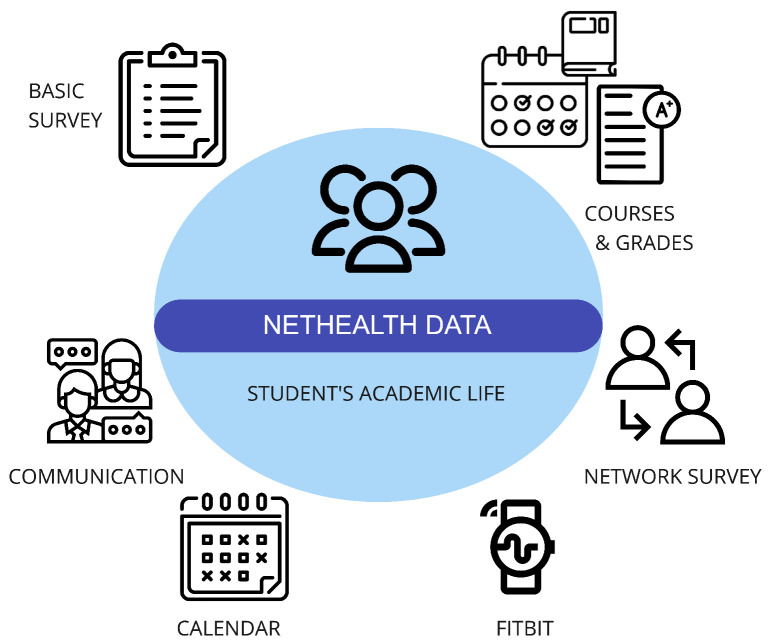
NetHealth dataset and its components.

The dataset encompasses various types of information, including social network data, physical activity data collected from Fitbit wearable devices (such as step counts, active minutes, heart rate, and sleep metrics), the administrative records of courses and grades from the ND Registrar’s Office, a calendar displaying weekly class schedules and breaks, and self-reported survey data covering aspects like physical and mental health, social-psychological states, personal preferences, self-reported behaviors, demographic information, and background traits (Figure 1).

It is important to note that the data collection process complied with Institutional Review Board (IRB) protocols, with the informed consent of all participating individuals. However, some data are not publicly shared due to privacy concerns.

In this study, we specifically focused on distinct sub-datasets within the collected data, including wearable data containing fitness and sleep measurements and self-reported survey data. Additionally, we combined all semester’s information and imputed the missing values for expanded data, which will be explained in the data preprocessing steps (Section 3.3). Since a large number of survey questions are collected, we are focusing on only some selected ones in this study’s context. For clarity in navigating the parameter space, we provide detailed feature information in Table 1. Our feature selection aligns with our previous study [19], except for the grade dataset, as it falls outside the scope of the current study. Similarly, mental health features are excluded, as they serve as target values. It is essential to note that, unlike Table 1 in the previous study [19], the features in the current analysis are not categorized by semester number since we utilize information spanning all semesters.

#### 3.2.1. Sensing Streams

Within the scope of our study, we have directed our attention to specific sub-datasets within the broader dataset. These sub-datasets encompass wearable data, capturing essential fitness and sleep measurements through Fitbit devices. Furthermore, we delved into the self-reported survey data, which offer a multifaceted view of students’ well-being. We did not utilize other data information, such as social network and communication data, since they require different methodologies, such as graph neural networks, to obtain meaningful results. We will consider them in our future studies.

#### 3.2.2. Predictors

Our research focuses on the assessment and prediction of three mental health indicators—stress, anxiety, and depression—similarly to the DASS [20]. To unravel the interplays between these mental health conditions and an array of predictor variables, we consider a diverse set of factors, encompassing physical activity levels, sleep patterns, demographic characteristics, and self-reported behaviors.

#### 3.2.3. Target Values

Even though we focus on DAS parameters, our dataset does not explicitly contain DASS values collected in a single questionnaire, as performed for DASS-21 or DASS-42; instead, we have separately collected values for depression, anxiety, and stress. For simplicity, we will use the term ‘DAS’ to represent all three target variables. In the following, we describe the target values and their characteristics specific to the current dataset.

- Depression
  Depression is a pervasive mental health condition marked by persistent and deep-seated feelings of sadness, hopelessness, and diminished interest or pleasure in once-enjoyable activities, leading to substantial impairment in daily functioning and overall quality of life [1,21]. NetHealth contains two different survey responses for depression such as ‘CES-D’ https://www.apa.org/pi/about/publications/caregivers/practice-settings/assessment/tools/depression-scale (accessed on 17 January 2024) and ‘BDI’ https://www.apa.org/pi/about/publications/caregivers/practice-settings/assessment/tools/beck-depression (accessed on 17 January 2024). Additionally, we have continuous scores ranging from 0 to 60 for CES-D and from 0 to 63 for BDI, with higher scores indicating greater depressive symptoms. For CES-D, there is also a grouped version ranging from 0 to 1, representing not depressed and depressed. For BDI, the grouped version ranges from 0 to 3, representing minimal, mild, moderate, and severe levels of depression. Throughout the study, we employed the BDI with its corresponding classification levels.
- Anxiety
  Anxiety is a prolonged state of excessive worry and fear about future events, characterized by heightened alertness and unease; while it can be a normal response to stress, overwhelming and impairing forms may indicate an anxiety disorder [1,22]. Similar to depression values, NetHealth also contains two different survey responses for anxiety: ‘STAI’ https://www.apa.org/pi/about/publications/caregivers/practice-settings/assessment/tools/trait-state (accessed on 17 January 2024) and ‘BAI’ https://en.wikipedia.org/wiki/Beck_Depression_Inventory (accessed on 17 January 2024). We have continuous scores ranging from 20 to 80 for STAI and from 0 to 63 for BAI, where higher scores indicate greater anxiety. STAI has a grouped version ranging from 0 to 1, representing not anxious and anxious, respectively. On the other hand, BAI ranges from 0 to 2, representing low, moderate, and severe anxiety levels. In the scope of this study, we utilized the BAI with its corresponding levels.
- Stress
  Stress is a physiological and psychological response to perceived threats, encompassing external and internal stressors, triggering the body’s “fight or flight” response; while acute stress can be adaptive, chronic stress, persisting over time, can negatively impact both physical and mental health, contributing to conditions like anxiety and depression [1,23]. In NetHealth, we have only ‘PSS’ https://www.das.nh.gov/wellness/docs/percieved%20stress%20scale.pdf (accessed on 17 January 2024). We are only provided with its Likert-scale version ranging from 0 to 4, corresponding to never through very often. Given the absence of alternative options for the stress target, we utilized a class-based scale for the entire study. We made choices regarding the survey and value types for other target variables (raw continuous value or classes) by aligning them with the response type available for stress. This decision was made to ensure coherence among target variables despite the differing class scale ranges.

### 3.3. Missing Data

In the context of our study, spanning 8 semesters of data, we encountered challenges as certain questions were not consistently addressed or answered across all semesters. Despite having rich data over the semesters, the absence of commonalities prompted the need for a comprehensive approach to handling missing data.

To manage missing data effectively, we adopted the Multiple Imputation by Chained Equations (MICE) method [24]. MICE provides a systematic framework for filling in data gaps by generating educated estimates based on the available data. The process involves iteratively imputing missing values for each variable using conditional distributions. In simpler terms, MICE breaks down the imputation process into a series of steps, where each variable’s missing values are estimated based on the observed values of other variables. This iterative process continues until convergence is achieved, resulting in multiple complete datasets.

Upon examination, we identified that missing data from wearables were relatively minimal. However, substantial amounts of missing values were observed in survey data. For certain variables, such as gender and related survey questions, which were not consistently posed every semester, we addressed the issue by replicating a single collected response across all missing columns for each individual throughout different semesters. On the other hand, we employed MICE to fill in the missing values for missing values related to aspects like sleep, health, and mental health features, ensuring a more holistic dataset for our analysis. At the end of this process, we ended up with 310,967 rows and 74 columns.

### 3.4. Data Preprocessing for Time-Based Methods

To encounter time aspect and temporal dependencies within the data, we employed a time-lagged version of the data where each data point is associated with the previous data point. Even though LSTM has its own time dependency, in our previous study on another dataset [10], we found that LSTM on time-lagged data further improved model performance.

Consider a mental health parameter, like depression, anxiety, or stress (DAS) at a given time ‘t’. To be able to create a lagged version of DAS, we present the DAS parameter as follows:

(1) DAS(t)=[DAS(t−n),DAS(t−(n−1)),…,DAS(t−1),DAS(t)]

Here, DAS(t) represents one of the mental health parameters (e.g., depression level) at a time ‘t’, ranging from, for instance, 0 to 4 for severity. DAS(t−n),DAS(t−(n−1)), …, DAS(t−1),DAS(t) represent the individual measurements of the mental health parameter at different time points within a specified time window. The ‘n’ in this context signifies the size of the time window, indicating the number of past measurements considered when assessing the mental health parameter.

In our study, we employed various look-back (n) sizes, such as 1, 7, 15, and 30, a semester to consider daily, weekly, fortnightly, monthly, and semesterly patterns in mental health data.

### 3.5. Models and Evaluation Metrics

We employed traditional RF, XGBoost, LSTM, and their lagged versions as prediction/regression models. Since one of our goals is to show the effect of MTL, we employed multitask versions of baselines (RF, XGBoost, LSTM) and their lagged versions. Once we found the best-performing look-back and look-up combination with the baseline’s time-lagged version, we employed the same combination, which is 1-day look-up and 15-day look-back, for the time-lagged version of the multitask.

Since all of our tasks are regression-based for MTL, we employed multi-output-based multitask learning. Multitask learning and multi-output learning differ primarily because output variables in multi-output learning often share the same training data or features. In contrast, separate tasks may be learned in MTL on different training sets or features. Multitask supervised learning becomes multi-label learning or multi-output regression when multiple tasks share the training set [6,25]. Instead of running algorithms over the same data for different task predictions, by employing MTL, we aim to run each method once to measure their performance on different tasks.

The RF algorithm is employed due to its performances in the literature [10,26]; XGBoost is employed as it is indicated to perform better compared to deep-learning techniques and even better once combined with deep-learning compared to the alone version [9,27]; and LSTM is employed as it inherently considers the time aspects of data. The hyper-parameters for each algorithm are provided in Table A1.

We also have their lagged versions for 1,7,15,30,90,180 days look-back to consider the contribution of past data points and 1,7,15,30 days lookup to understand up to how many days we can reach reasonable errors and which one’s performance is better.

**Figure 2:**
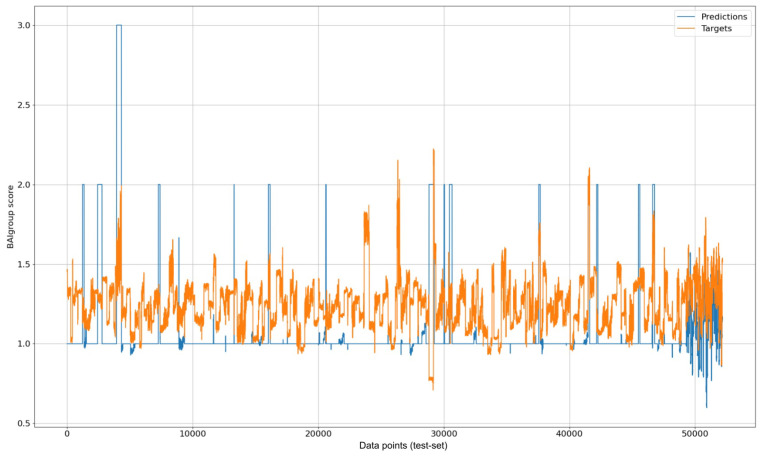
Visualization of multitask LSTM prediction results for anxiety over test data.

We used MAE as an error metric since we did not encounter a sign difference between the target and predicted variable (an example of this visualization can be seen in Figure 2). It belongs to the multitask-LSTM-anxiety case’s visualization. The X axis represents the indexes corresponding to the data points in the testing dataset. Furthermore, to prevent over-fitting, we split the dataset into 5, 2, and 2 semesters for train, validation, and testing, respectively.

Please note that while the target variables in the original dataset were discrete, the MICE application resulted in obtaining continuous values for missing target entries. Considering our expertise, especially in the context of psychological questionnaires, we refrained from categorizing these values into specific classes. Instead, we chose to utilize models that align with the nature of the dataset, prioritizing prediction over classification.

Additionally, due to the same consideration of utilizing prediction models, there are instances where the predicted values fall outside the range of the original target values. Despite this, we opted not to remove such instances. Our rationale is rooted in the nature of our predictive modeling approach, where the goal is to estimate numerical values without imposing constraints based on predefined classes.

## 4. Quantification of Biomarkers Related to DAS

We employed SHAP (Shapley Additive Explanations) values [28] to assess the influence of each feature on the primary model. Drawing inspiration from the game theory’s Shapley value, this technique gauges the individual contributions of each feature towards the model’s outcomes for interpreting machine learning models.

**Figure 3:**
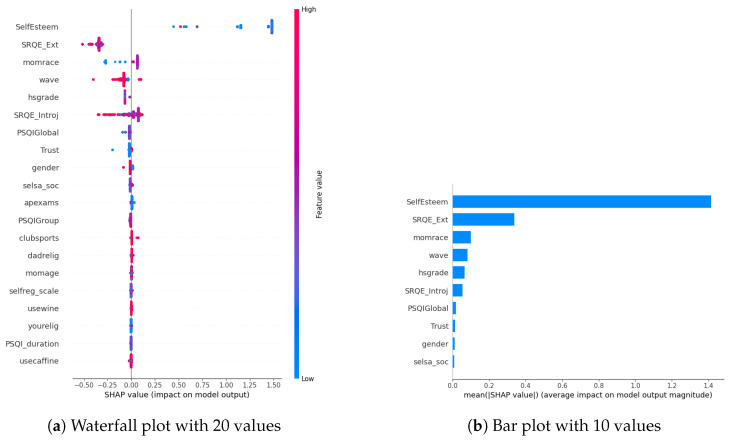
Depression biomarkers.

**Figure 4:**
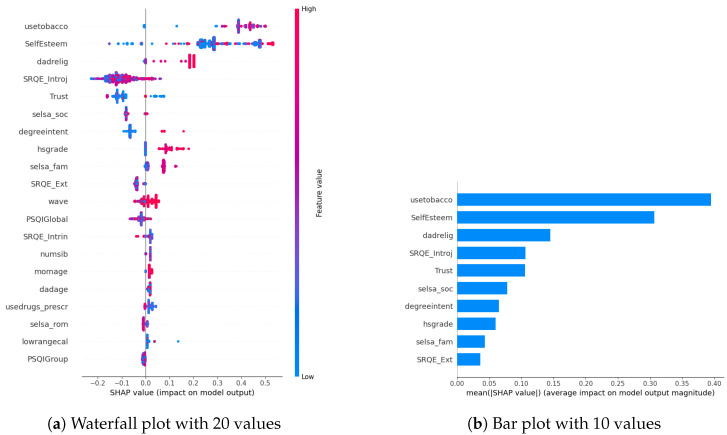
Anxiety biomarkers.

**Figure 5:**
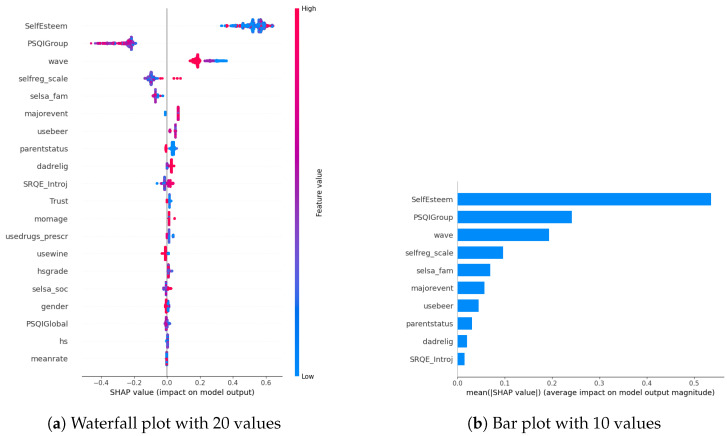
Stress biomarkers.

In our examination of the mental well-being model using SHAP, we assessed the relative impact of the top 20 features based on mean absolute SHAP values for each target variable separately (Figure 3a, Figure 4a and Figure 5a). The key contributors to the model’s predictive power included SRQE_introj (introjective self-regulation), SRQE_Ext (external self-regulation), SelfEsteem (on the whole, I am satisfied with myself), PSQI (sleep quality index), trust (most people can be trusted), selsa (loneliness) along with other education and origin-related parameters. These findings highlight the crucial role of these factors in influencing the model’s predictions related to mental well-being. Since the effect of parameters becomes lower and lower at some point, to facilitate understanding, we only plot the first ten parameters in Figure 3b, Figure 4b and Figure 5b. We answered our first research question (RQ1) by providing SHAP values.

Our prior study concentrated on the well-being of office workers, specifically examining stress, anxiety, and mood (positive and negative affect); our research identified personality factors such as extraversion, agreeableness, conscientiousness, neuroticism, and openness (Big-5 inventory) as among the most crucial contributors [10]. However, within the context of the NetHealth dataset, despite collecting these personality values, none of them emerged among the top 20 results according to SHAP analysis. We attribute this phenomenon to the specificity of the dataset, revealing that other factors beyond personality traits play a much more significant role in influencing outcomes.

Furthermore, in [29], researchers focused on adolescents’ self-regulation and mental health relation. Engaging in exploration, formulation, implementation, and assessment is found to be positively associated with the mental health of adolescent boys and girls. This suggests that individuals who actively explore various options in their environment, consider alternatives, and maintain focus on tasks instead of getting distracted tend to evaluate events more positively. Consequently, this approach is linked to lower cognitive distortion, enhancing psychological functioning and higher self-esteem. These results also correlate with our findings about the self-esteem parameter (in stress SHAP results).

Different sleep sub-parameters, such as overall quality value in the continuous value between 0 and 21 or its classified version under several classes indicating sleep quality or sleep duration, are observed in all of the target values’ SHAP parameters. This aligns with the findings in the literature. In [30], researchers found that greater improvements in sleep quality lead to more significant improvements in mental health. The discussion emphasizes the trans-diagnostic nature of sleep as a treatment target, noting that the benefits extend across various populations, whether individuals have clinically defined mental health difficulties or non-clinical experiences. Additionally, targeting sleep is seen as a preventive measure, potentially limiting the risk of developing or exacerbating mental health difficulties in the future. This finding also aligns with our previous study on office workers [10], where sleep-related parameters were found commonly among all the targets’ most important parameters.

Among the common parameters of all target variables, we found loneliness and its derivatives, such as romantic, family, or social loneliness. In addition to loneliness, the trust parameter, which indicates the perspective of the participant over the other people, whether they are trustable or not, is observed in the depression and stress target values SHAP list. This study [31] investigated how loneliness and trust issues, both in personal relationships and institutions, impacts stress among expatriates during the early COVID-19 pandemic. It found a direct relationship between perceived stress, loneliness, and trust levels. Low trust, especially in interpersonal relationships, led to increased loneliness. Conversely, higher interpersonal trust was linked to lower perceived stress. Institutional trust had a more pronounced impact on stress than loneliness. The study highlighted the evolving trust dynamics during the pandemic, including strained relationships with academic institutions. Overall, the findings emphasized the interconnectedness of trust, loneliness, and mental well-being among expatriates during challenging times.

In our SHAP values analysis, we observed notable contributions from parameters like “wave”, underscoring the importance of semester-specific data in predicting target values. We can validate temporal importance with time-based algorithm performance improvements in Section 5. It is important to note that the current study omits personalization, leaving this aspect for consideration in future research studies. In this way, we answered our second research question (RQ2).

### Biomarkers Related to DAS via Only Wearable Parameters

**Figure 6:**
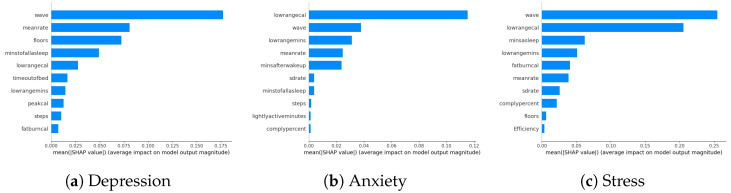
DAS biomarkers using only the parameters related to wearables.

Our study examines the impact of survey questions and wearables on model performance in predicting DAS. While wearables are not identified as the most crucial factors in SHAP analyses, their specific ranking is explored by excluding survey parameters in this section. The model performance results indicate a slight increase in errors compared to including all parameters, suggesting that survey parameters contribute marginally more to model performance. Despite wearables being positioned towards the end of the parameter space, their influence is overshadowed by the minor contributions of other survey parameters. Detailed parameter contributions exclusively focusing on wearables are visually presented in Figure 6a–c, illustrating the role of wearable-related factors in the model predictions. The ranking of wearable-related factors in influencing the model predictions is evident from these visualizations.

## 5. Conventional, Multitask, Time-Based, and Multitask Time-Based DAS Predictions

We are dealing with three target variables: depression on a scale of 0 to 3, anxiety on a scale of 0 to 2, and stress on a scale of 0 to 4. We aim to predict these target values using RF, XGBoost, and LSTM. Additionally, we aim to empirically assess the impact of a lagged dataset on performance and investigate whether multitasking alone or in combination with the lagged version influences outcomes (RQ3, RQ4).

The comprehensive outcomes are outlined in Table 2, with additional insights into time-based performances accessible in Table 3. Despite their minimal impact on the ultimate performance enhancement, these details afford a broad perspective on the consequences of diverse look-back and look-up combinations.

The baseline and MTL performances are closely aligned, with MTL showing no significant improvement. RF and XGBoost results also exhibit a minimal difference in both scenarios. Notably, incorporating the temporal aspect has led to substantial performance enhancements, with the highest outcome achieved using RF with a 15-day look-back and 1-day lookup. While Table 2 outlines the time-based results for a 1-day lookup, Table 3 encompasses various combinations of look-backs and look-ups. In addition, when considering a longer look-back period in the time-based method, such as comparing 15 days to 1 month or 1 month to 1 semester, despite the increased computational and time burden, we did not observe any performance improvement. In the pursuit to refine time-based performances, we implemented the multitask version on top of time-based methods. This strategy yielded improved outcomes for depression and stress target variables, although it did not enhance the anxiety target performances. RF and XGBoost performances remained closely aligned. An interesting observation was the consistently poorer performances with LSTM across all cases compared to RF and XGBoost. However, the difference is not too high. Since LSTM intrinsically handles the time aspect of data and we observe that there is an improvement when we consider lagged data, this might be due to the usage of a simple LSTM architecture with only one layer.

A closer look at Table 3 reveals a general trend of increasing error values (indicating worse performance) from top to bottom as we compare each algorithm with its counterpart in different combinations. However, this trend is not consistently observed in all cases. For instance, the performance of LSTM in the 7-day look-up and 1-semester look-back combination is improved when employing the 15-day look-up and 15-day look-back combination. Therefore, a consistent pattern that applies to all cases and algorithms cannot be conclusively determined. Interestingly, longer look-back periods did not result in performance improvements. Implementing MTL on time-based methods improved outcomes for depression and stress but did not enhance anxiety target performances. LSTM consistently exhibited poorer performances across all cases compared to RF and XGBoost. Analyzing Table 3 reveals an overall trend of increasing error values from top to bottom, which is inconsistent across all cases and algorithms. For example, the LSTM performance improved in the 7-day look-up and 1-semester look-back combination when employing the 15-day look-up and 15-day look-back combination, suggesting a lack of a universal pattern.

To the best of our knowledge, our study is the first to incorporate the time-based aspect of data among the studies utilizing NetHealth. Unlike studies solely focusing on one target variable, we selected three related variables appropriately termed DAS in the literature. Furthermore, in addition to the time-based improvement, we utilized MTL. Since all three tasks involve regression, we approached multitasking as a multi-output regression problem. This approach allowed us to fairly compare algorithm performances only while concentrating on their type changes rather than their architectures.

## 6. Conclusions and Future Work

We focused on predicting mental well-being factors, including depression, stress, and anxiety, using the NetHealth dataset from college students. Addressing four research questions, we explored digital biomarkers’ impact on these factors (RQ1), their alignment with conventional psychology literature (RQ2), the time-based performance of applied methods (RQ3), and since the target variables are correlated, we wanted to explore further performance improvements while implementing specific methods designed for this purpose such as MTL (RQ4).

Our findings revealed interesting modality rankings for each target variable, aligning with conventional psychology literature for each parameter. Additionally, we validated our results by comparing them with paper-based studies that lacked wearable device usage. We ended up with no parameter coming from wearable on the top 20 variables. That means their contributions to the final model performance are lower than the presented survey parameters in the SHAP figures. Device-measured modalities aligned with traditional studies, and we observed improved prediction performances when considering the temporal aspect of the data. We further improved by employing the multitasking technique while considering the time aspect of data. The updated analysis of mental health multitask results indicates that baseline and MTL performances align closely, with no significant improvement observed in the multitask scenario. RF and XGBoost results exhibit minimal differences, and substantial performance enhancements are achieved by incorporating the temporal aspect, particularly with RF using a 15-day look-back and 1-day lookup.

There are some limitations to this study. We observed that “egoid” is among the most important parameters, indicating that we may achieve further high performances by personalization. However, we did not encounter personalization in the context of the current study. We are planning to consider it in future studies.

## Figures and Tables

**Table 1:** Details of the parameters in the dataset.

Dataset	Measured Values
Wearable data (Activity)	complypercent (percent minutes using Fitbit), meanrate (mean heart rate), sdrate (st. dev. heart rate), steps, floors, sedentaryminutes, lightlyactiveminutes, fairlyactiveminutes, veryactiveminutes, lowrangemins, fatburnmins, cardiomins, peakmins, lowrangecal, fatburncal, cardiocal, peakcal
Wearable data (Sleep)	timetobed (time went to bed), timeoutofbed (time out of bed), bedtimedur (minutues in bed in minutes), minstofallasleep (minutes to fall asleep), minsafterwakeup (minutes in bed after waking), minsasleep (minutes asleep), minsawake (minutes awake during sleep period), Efficiency (minsasleep/(minsasleep + minsawake))
Survey data (Bad habits)	usetobacco (used tobacco), usebeer (drank beer), usewine (drank wine or liquor), usedrugs (used rec drugs like marij. or cocaine), usedrugs_prescr (used presc. drugs not prescribed), usecaffine (drank caffenated drinks)
Survey data (BigFive/Personal inventory)	Extraversion, Agreeableness, Conscientiousness, Neuroticism, Openness
Survey data (Education)	hs (high school type), hssex (high school sex composition), hsgrade (high school average grade), apexams (# of hs ap exams), degreeintent (highest intended degree), hrswork (paid hours senior year), ndfirst (Notre Dame first choice of applied colleges?)
Survey data (Exercise)	hsclubrc (club activities), exercise (excersise), clubsports (play club, intramural or rec sports), varsitysports (play varsity sports), swimming (swim), Dieting (special type of diet), PhysicalDisability (physical disability)
Survey data (Health)	SelfEsteem (on the whole, I am satisfied with myself), Trust (most people can be trusted), SRQE_Ext (external self-regulation (exercise)), SRQE_Introj (introjective self-regulation (exercise)), SRQE_Ident(identified self-regulation (exercise), SelfEff_exercise_scale (when i am feeling tired), SelfEff_diet_scale (self_efficacy score (diet items)), selfreg_scale (i have trouble making plans to help me reach my goals)
Survey data (Origin)	momdec (is your mother deceased?), momusa (was mother born outside usa?), daddec (is your dad deceased?), dadusa (was your dad born outside usa?), living together or divorced/living apart), dadage (father’s age), momage (mom’s age), numsib (number of siblings), birthorder (which # in birth order are you?), parentincome (parent’s total income last year), parenteduc (combined parent education), momrace (mother’s race), dadrace (father’s race), momrelig (mother’s religious preference), dadrelig (father’s religious preference), yourelig (your religious prefence)
Survey data (Personal info)	selsa_rom (romantic loneliness), selsa_fam (family loneliness), selsa_soc (social loneliness)
Survey data (Sex)	gender
Survey data (Sleep)	PSQI_duration (computed time in bed), PSQIGlobal (PSQI total score), PSQIGroup (PSQI two categories), MEQTotal (MEQ (chronotype) score - high score morning person), MEQGroup (MEQ (chronotype) groups)

**Table 2:** Model prediction performances for DAS, presented in terms of MAE, where bold indicates the best performance for each type.

Type		Method	Depression	Anxiety	Stress
Baseline		RF	1.1804	**0.1277**	0.2742
		XGBoost	**1.1537**	0.1588	**0.2722**
		LSTM	1.7342	0.1487	0.5199
Multitask		RF	**1.1600**	0.1348	**0.2794**
		XGBoost	1.1771	**0.1347**	0.2798
		LSTM	1.7181	0.2584	0.6029
Time-based baseline (1 day look-up)	1 day look back	RF	0.0198	0.0047	0.0314
		XGBoost	0.0433	0.0060	0.0264
		LSTM	0.0379	0.0221	0.0653
	7 days look back	RF	0.0198	0.0036	0.0230
		XGBoost	0.0361	0.0060	0.0278
		LSTM	0.0373	0.0178	0.0646
	15 days look back	RF	**0.0194**	**0.0047**	**0.0228**
		XGBoost	0.0399	0.0072	0.0289
		LSTM	0.0424	0.0134	0.0703
	1 month look back	RF	0.0198	0.0047	0.0233
		XGBoost	0.0433	0.0059	0.0292
		LSTM	0.0462	0.0220	0.0741
	1 semester look back	RF	0.0234	0.0048	**0.0220**
		XGBoost	0.0371	0.0059	0.0270
		LSTM	0.0738	0.0286	0.0792
Time-based multitask		RF	**0.0049**	0.0131	0.0166
		XGBoost	0.0070	**0.0083**	**0.0091**
		LSTM	0.0319	0.0352	0.0381

**Table 3:** Further results of the time-based predictions.

Type	Time	Details	Method	Depression	Anxiety	Stress
Time-based	7 days lookup	7 days look back	RF	0.0528	0.0105	0.0436
			XGBoost	0.1324	0.0190	0.0455
			LSTM	0.0392	0.0109	0.0704
		15 days look back	RF	0.0563	0.0114	0.0397
			XGBoost	0.1339	0.0200	0.0426
			LSTM	0.0492	0.0188	0.0739
		1 month look back	RF	0.0535	0.0097	0.0442
			XGBoost	0.1298	0.0187	0.0448
			LSTM	0.0389	0.0150	0.0721
		1 semester look back	RF	0.0774	0.0097	0.0407
			XGBoost	0.1305	0.0182	0.0426
			LSTM	0.0993	0.0322	0.1508
	15 days lookup	15 days look back	RF	0.1082	0.0192	0.0618
			XGBoost	0.2426	0.0368	0.0648
			LSTM	0.0905	0.0142	0.0759
		1 month look back	RF	0.1091	0.0185	0.0662
			XGBoost	0.2409	0.0359	0.0691
			LSTM	0.0608	0.0281	0.1524
		1 semester look back	RF	0.1430	0.0196	0.0670
			XGBoost	0.2394	0.0355	0.0641
			LSTM	0.1265	0.0353	0.1074
	1 month lookup	1 month look back	RF	0.1955	0.0314	0.1055
			XGBoost	0.4281	0.0644	0.1009
			LSTM	0.0838	0.0361	0.0794
		1 semester look back	RF	0.2581	0.0322	0.1113
			XGBoost	0.4279	0.0640	0.0974
			LSTM	0.2443	0.0586	0.1596

## Data Availability

NetHealth dataset is available at https://sites.nd.edu/nethealth/ (accessed on 27 January 2024).

## Appendix A

**Table A1:** Hyper-parameters for different algorithms predicting each mental-health parameter.

Algorithm	Hyperparameters
RF-Depression	(‘max depth’, 5), (‘min samples leaf’, 1), (‘min samples split’, 2), (‘n estimators’, 10)
RF-Anxiety	(‘max depth’, 5), (‘min samples leaf’, 2), (‘min samples split’, 3), (‘n estimators’, 6)
RF-Stress	(‘max depth’, 4), (‘min samples leaf’, 1), (‘min samples split’, 10), (‘n estimators’, 5)
XGBoost-Depression	(‘alpha’, 1), (‘colsample bytree’, 0.7333635223098999), (‘gamma’, 1), (‘learning rate’, 0.058336569586572316),
	(‘max depth’, 8), (‘min child weight’, 3), (‘n estimators’, 82), (‘subsample’, 0.641372976280757)
XGBoost-Anxiety	(‘alpha’, 0), (‘colsample bytree’, 0.510066209080432), (‘gamma’, 0), (‘learning rate’, 0.04359820016477877),
	(‘max depth’, 7), (‘min child weight’, 8), (‘n estimators’, 70), (‘subsample’, 0.9080823751699505)
XGBoost-Stress	(‘alpha’, 0), (‘colsample bytree’, 1.0), (‘gamma’, 0), (‘learning rate’, 0.2993), (‘max depth’, 3), (‘min child weight’, 6),
	(‘n estimators’, 157), (‘subsample’, 0.5)
LSTM	25 layered LSTM with epochs 10, batch size 72, Adam optimizer with learning rate 0.001, hyperbolic tangent as an activation function,
	MAE as a loss function
RF-MTL	(‘max depth’, 6), (‘min samples leaf’, 1), (‘min samples split’, 6), (‘n estimators’, 5)
XGBoost-MTL	(‘alpha’, 0), (‘colsample bytree’, 0.8530567994101286), (‘gamma’, 1), (‘learning rate’, 0.07023334252979543), (‘max depth’, 4),
	(‘min child weight’, 7), (‘n estimators’, 124), (‘subsample’, 0.9930831348301362)
LSTM-MTL	32 layered LSTM with epochs 10, batch size 8, Adam optimizer with learning rate 0.001, hyperbolic tangent as an activation function,
	MAE as a loss function
